# Effect of Systemic Intraoperative Heparin Use on Upper Extremity Arteriovenous Fistula Patency in End-Stage Renal Disease Patients

**DOI:** 10.1155/2021/2965743

**Published:** 2021-10-31

**Authors:** Morwan Bahi

**Affiliations:** MBChB, Department of Vascular Surgery, Wellington Hospital, Wellington, New Zealand

## Abstract

The formation of the arteriovenous fistula is an important method of vascular access for patients with end-stage renal disease (ESRD). This allows renal filtration resulting in improved life quality and expectancy for ESRD patients. The biggest drawback to arteriovenous fistula formation is thrombosis, which can occur at an early or delayed stage. One suggested method of reducing postoperative arteriovenous fistula thrombosis rates is the administration of intraoperative systemic heparin. Heparin use in this context is debated, and there is currently no consensus on its use. There are a number of small randomised control studies trialling use of heparin but no large systematic trials. In this report, we collate existing evidence in the form of a review article and attempt to extrapolate a consensus of the evidence.

## 1. Introduction

End-stage renal disease (ESRD) is increasing in prevalence worldwide [1]. ESRD patients require a method of access to allow hemodialysis or peritoneal dialysis [2, 3]. Options include temporary venous lines, intraperitoneal catheters, or formation of the arteriovenous fistula (AVF) [1–3]. AVF is an important method due to its longevity and lower rate of complications [1–3]. This involves the creation of a continuous anastomosis between a suitable artery and vein, typically in the upper limb, to allow development of a suitable access site [3].

The most common AVF types are radiocephalic, brachiocephalic, and brachiobasilic fistulas [4]. In suitable patients, radiocephalic AVF is first-line as it preserves more proximal vessels should further AVF formation be required in future [4]. Brachiocephalic fistulas are preferred due to the larger diameter of the brachial artery (in comparison to radial) allowing for better outcomes [3, 4]. There are other methods of access including arteriovenous grafts and saphenous loop fistulas, but these will not be discussed here. Selection of appropriate vessels for AVF formation depends on physical examination of the limb as well as preoperative imaging (duplex ultrasound) with vessels >2-3 mm in diameter deemed suitable [3, 4].

There are a number of complications associated with AVF formation including bleeding, infection, limb ischaemia, failure to mature, and thrombosis [1, 3, 4]. Thrombosis can develop in the early postoperative phase or can be delayed [1]. As a method of reducing thrombosis rates, use of intraoperative anticoagulation with systemic heparin has been suggested [1, 2]. Heparin acts as an anticoagulant preventing formation and/or extension of the intravascular clot [1, 2]. However, current evidence on heparin use is lacking and contradictory in nature [2].

This review will focus on the rate of patency following upper extremity AVF formation with regard to intraoperative systemic heparin use. Rates of bleeding and haematoma formation will also be discussed. We will review the existing literature and summarise outcomes.

## 2. Methods

### 2.1. Search Strategy

Literature search was performed using Medline, PubMed, and Embase databases and included all publications up until June 2021. Randomised control trials (RCTs) were included. The search strategy aimed to include studies comparing upper extremity AVF outcomes with or without the use of intraoperative systemic heparin in adult ESRD patients.

Search terms included fistula/arteriovenous fistula/vascular access/dialysis/hemofiltration/heparin/anticoagulation/fistula thrombosis/vascular access surgery.

### 2.2. Inclusion Criteria

Studies were eligible if they

- Were RCTs in English language
- Included adult (>18 years) ESRD patients only
- Investigated outcomes for upper limb AVF only (brachiocephalic, brachiobasilic, and radiocephalic fistulas); studies describing other forms of AVF or other forms of vascular access were not included
- Compared use of heparin in case and control groups and no other anticoagulants or antiplatelet agents

### 2.3. Outcomes

- The primary outcome will be AVF patency rate with relation to intraoperative systemic heparin use
- The secondary outcome will be postoperative bleeding and haematoma formation

### 2.4. Data Analysis

For the meta-analysis, data were entered into and analysed in R statistical software (R Foundation for Statistical Computing, Vienna, Austria). A random-effect meta-analysis of dichotomous outcomes of AVF patency was presented using risk ratios with 95% confidence intervals.

## 3. Results

### 3.1. Literature Search

**Figure 1:**
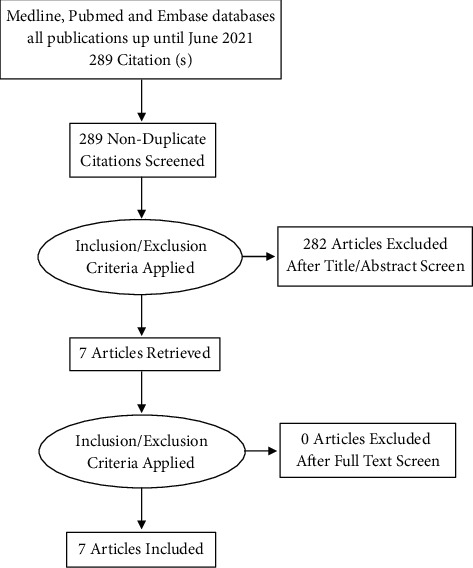
PRISMA flow diagram summarising the search methodology.

Literature search as detailed in Section 2 identified a pool of 289 studies. Of those, 282 were not eligible based on inclusion criteria. Seven studies (RCTs) were included following full-text review of all studies (see Figure 1).

### 3.2. Summary of Included Studies (See Table 1)

Ravari et al. [5] compared the use of heparin with no heparin. Only brachiocephalic and radiocephalic AVFs were included. Patients were randomised, but the method of randomisation is not described. Case patients received 5000 IU heparin immediately prior to arteriovenous anastomosis. AVF patency was assessed at the end of case and at 2 weeks postoperatively. The study included 85 brachiocephalic fistulas (36 cases and 49 controls) and 113 radiocephalic fistulas (66 cases and 47 controls). This study did not report a statistical difference in AVF patency immediately postoperatively; however, it reported improved patency at 2 weeks postoperatively (85% with heparin vs. 74% without, *p*=0.046). This study did not report on the surgical technique or surgeon expertise. The study reported no statistically significant difference in haematoma rates between case and control groups.

Ebrahimifard [6] performed a single-blind randomised study comparing heparin to no anticoagulation. The case group received 5000 IU heparin prior to clamping the artery. The study included 50 patients. The method of randomisation was not described. Surgeon factors were not discussed. Fistulas were assessed at 24 hours and 6 weeks. The heparin group experienced no early postoperative thrombosis, while the control group experienced 7 cases of early postoperative thrombosis (*P*=0.004). Late thrombosis was observed in one patient in the heparin group compared to 2 in the control group without statistical significance (*P*=0.22). There was no statistical significance for haematoma formation or bleeding between the groups.

Aimanan et al. [1] conducted a double-blind RCT focusing on radiocephalic AVF outcomes in a sample of 90 patients comparing heparin (80 IU/kg) to placebo (saline). Randomisation took place through patient selection of a closed envelope containing information for heparin or placebo. Surgeons performing AVF formation were described as senior (>100 previous AVF formations) or junior (<100 cases). There was no statistical difference in surgeon expertise between the 2 groups. AVF patency was assessed at 1 week and 4 weeks following formation. At one week, AVF thrombus was present in 4 patients in the heparin group compared with 11 in the saline group. This was compared with 4 and 12, respectively, at the 4-week review. The difference was reported as being statistically significant (*P*=0.03). The study reported no statistically significant difference in haematoma risk between the case and control groups (*P*=0.53).

Mozafar et al. [7] investigated the effect of heparin on early postoperative upper limb AVF patency. Randomisation method is not described. Case patients received 100 IU/kg systemic heparin prior to anastomosis. All procedures were performed by a single surgeon. At 24 hours postoperatively, 6 control patients had a nonfunctioning fistula comparing to zero in the heparin group (*P*=0.028). No details on postoperative complications were provided.

D'yala et al. [8] conducted a single-blind RCT investigating the effect of heparin on upper limb AVF patency. Randomisation method was not described. Case patients received 5000 IU systemic heparin prior to arterial clamping. Control patients received no anticoagulation. The study included 115 patients. All procedures were performed by the same surgeon. Thirteen patients experienced postoperative bleeding in the recovery area postoperatively in the heparin group compared to one case of bleeding in the control group (*P*=0.008). The 30-day patency was 84% in the heparin group compared to 86% in the control group (*P*=0.79). Patency at 3 months for both groups was 68% (*P*=0.99).

Bhomi et al. [9] conducted a single-blind RCT where case patients received 5000 IU systemic heparin, while control patients received no anticoagulation. Fifty patients were included in the study. Early postoperative bleeding was more prevalent in the heparin group (*P* < 0.01). Six-week AVF patency was 96% in the heparin group compared with 92% in the control group (*P*=0.46). The authors concluded a higher risk of bleeding with heparin with no statistically significant improvement in AVF patency.

Wang et al. [10] conducted a double-blinded RCT. Randomisation method was not described. Case patients received 75 IU/kg systemic heparin through the anesthesiologist 2 minutes prior to clamping the artery. A total of 53 patients were included. The outcome was day 30 AVF patency. This was 92% in the heparin group compared with 86% in the control group (*P*=0.65). There were 3 haematomas in the heparin group compared with one in the control group (*P*=0.61). The study concluded no difference in AVF patency or bleeding with the use of systemic heparin.

## 4. Discussion

Arteriovenous fistula formation remains an important method for vascular access in ESRD patients requiring hemodialysis. However, this method is not without its set of complications, namely, thrombosis. This article reviews available evidence (RCTs) on this topic. We reviewed 7 studies in this brief review with outcomes summarised in Table 1. A meta-analysis of dichotomous outcomes of AVF patency was performed.

### 4.1. Arteriovenous Fistula Patency

**Figure 2:**
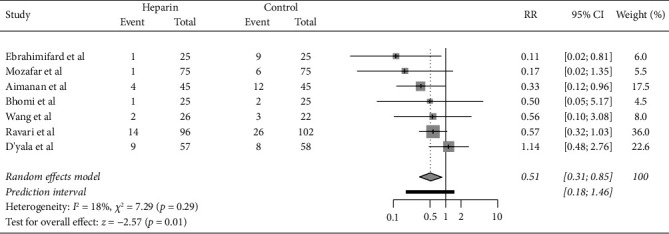
Forest plot suggesting benefit from intraoperative heparin use on the risk of loss of AVF patency.

Current evidence on heparin use to improve AVF patency is mixed. Four studies in this review have reported a statistically significant improvement in AVF patency with heparin use at the first follow-up, while 3 studies found no benefit. A meta-analysis of dichotomous outcomes across the 7 RCTs shows a statistically significant improvement in AVF patency with intraoperative heparin use as shown in Figure 2 (*P*=0.01). However, this is confounded by the temporal sequence between heparin half-life and time to follow-up reported in the RCTs. Heparin is only expected to last within the circulation for 1-2 hours, whereas most RCTs reported longer-term outcomes [2]. Only 2 RCTs assessed early AVF patency (24 hours post operatively) [6, 7]. Both found a statistically significant improvement in AVF patency. The remainder of the RCTs assessed longer-term outcomes (1 week [1], 30 days [8, 10], 2 weeks [5], and 6 weeks [9]). It is difficult to draw conclusions between heparin use and long-term outcomes given its short half-life of several hours. Furthermore, dosing of heparin was not standardised across trials as shown in Table 1. Four trials used 5000 IU for case patients; one trial used 75 IU/kg, one used 80 IU/kg, and one used 100 IU/kg. The trials were generally consistent in the administration of heparin prior to clamping the artery.

Four RCTs supporting heparin use included a total of 488 patients. The description of the specific methodology including the method of randomisation was lacking in all papers making the general quality relatively low, and it was not possible to assess a number of factors including bias and reproducibility. The Jaded score was generally low.

The 3 studies suggesting no benefit from heparin use included 218 patients. Again, the description of the study methodology and randomisation was extremely limited, and it was not possible to assess the quality or bias within trials. The Jaded score was again generally low.

### 4.2. Bleeding/Haematoma

Two studies reported statistically significant increased rates of bleeding or haematoma with systemic heparin use during AVF formation. These included 165 patients and were both RCTs. As discussed prior, study methodology was not detailed, and extrapolation of the findings was uncertain.

### 4.3. Type of Fistula

The included studies assessed outcomes in different types of fistulas. We only included RCTs assessing upper extremity fistula formation in this report. Two studies included patients undergoing radiocephalic fistula formation only, 2 studies included patients undergoing any upper extremity AVF, while 4 studies did not describe which type of upper extremity fistula was included (see Table 1).

This is problematic and a clear source of bias. Certain upper extremity AVFs such as radiocephalic AVF experience higher rates of failure [4]. This is postulated to be due to the smaller diameter of vessels and higher stenosis rate [4].

### 4.4. Surgeon Experience

Surgeon expertise is an important factor in postoperative outcomes and should always be considered when analysing surgical outcomes.

In 2 studies, the same surgeon performed all procedures which reduces the influence of surgeon factors on outcomes. One study stratified operating surgeon as junior or senior, while 5 studies did not comment on surgeon expertise or factors.

### 4.5. Summary and Recommendations

This review included 7 RCTs. Four studies supported the use of intraoperative heparin to improve AVF patency, while 3 studies found no statistically significant benefit. Two studies found a statistically significant increase in bleeding/haematoma postoperatively. A limited meta-analysis of dichotomous outcomes found a statistically significant improvement in AVF patency with heparin use (*P*=0.01). Our analysis compares with a previous meta-analysis by Smith et al. [2] published in 2015; however, we have the benefit of including 3 newer RCTs published since [1, 6, 7]. Both studies explored a primary outcome of AVF patency in the context of intraoperative heparin use. Smith et al. reported no statistically significant benefit from intraoperative heparin use on AVF patency. We report a statistically significant benefit from intraoperative heparin use from 7 RCTs and, however, identify methodological issues with most RCTs as well as the lack of temporal relation between intraoperative heparin use and long-term outcomes as most RCTs recorded AVF patency weeks to months postoperatively. We also provide a more descriptive analysis of included RCTs including comments on surgeon factors. This is missing from the earlier review by Smith et al. Both studies identify the need for further large RCTs to further study the topic.

In summary, it is difficult to extrapolate the results of the included trials due to their generally small sample size, limited description of the methodology, and variable analysis including heterogeneous fistula types, missing surgeon details, and incomplete reporting of outcomes in some instances. AVF patency was also assessed at different timeframes between trials ranging from 24 hours postoperatively to 3 months postoperatively with no standardised method of assessing patency.

## 5. Conclusion

End-stage renal disease is an increasing pathology worldwide. In order to improve the quality of life and longevity, patients require renal filtration which requires a method of vascular access. Arteriovenous fistula formation is the gold standard. The most significant complication is fistula thrombosis thus limiting its use. Use of intraoperative heparin has been suggested to improve AVF patency rates. Current evidence is somewhat supportive of heparin use for this purpose, but the evidence is inconclusive and of generally low quality. Ongoing larger, high-quality trials are required before use of heparin can be definitively recommended.

## Figures and Tables

**Table 1:** Summary of included studies including outcomes for patency and bleeding/haematoma.

Study	Type	Fistula type	Intervention (heparin)	Case/control sample size	Patency	Significance	Bleeding +/−haematoma
Ravari et al. (2008)	RCT	UL (BC/RC)	5000 IU	96/102	At 2 weeks: 85% heparin, 74% control	*P*=0.046	0 (both)
D'yala et al. (2008)	RCT	UL (NFD)	5000 IU	57/58	At 30 days: 84% heparin, 86% control At 3 months: 68% (both)	*P*=0.79 (30 days) *P*=0.99 (3 months)	13 (heparin) 1 (control)
Bhomi et al. (2008)	RCT	UL (NFD)	5000 IU	25/25	At 6 weeks: 96% heparin, 92% control	*P*=0.46	6 (heparin) 1 (control)
Wang et al. (2010)	RCT	UL (NFD)	75 IU/kg	28/25	At 30 days: 92% heparin, 86% control	*P*=0.65	3 (heparin) 1 (control)
Ebrahimifard (2015)	RCT	UL (RC)	5000 IU	25/25	Early (24 h): 100% heparin, 72% control Late (6 weeks): 96% heparin, 64% control	*P*=0.004	4 (heparin) 2 (control)
Aimanan et al. (2017)	RCT	UL (RC)	80 IU/kg	45/45	Early (1 week): 91% heparin, 76% control Late (4 weeks): 91% heparin, 73% control	*P*=0.05 (1 week) *P*=0.03 (4 weeks)	8 (heparin) 5 (control)
Mozafar et al. (2018)	RCT	UL (NFD)	100 IU/kg	75/75	At 24 hours: 100% heparin, 92% control	*P*=0.028	Not disclosed

UL: upper limb; NFD: not further described; BC: brachiocephalic; BB: brachiobasilic; RC: radiocephalic; IU: international units.

## Data Availability

The data used to support the findings of this study are available upon request to the author.
